# 41.4 DEPLOYMENT OF DEDICATED NURSING STAFF TO STIMULATE THE INITIATION OF CLOZAPINE. A CLUSTER-RANDOMIZED TRIAL

**DOI:** 10.1093/schbul/sby014.172

**Published:** 2018-04-01

**Authors:** Yvonne Van der Zalm, Raphael Schulte, Jan Bogers, Machteld Marcelis, Iris Sommer, Jean-Paul Selten

**Affiliations:** 1 Rivierduinen Psychiatric Institute; 2 Mental Health Service Noord-Holland Noord, Dutch Clozapine Collaboration Group; 3 Rivierduinen Centre for Mental Health / Dutch Clozapine Collaboration Group; 4 Maastricht University Medical Center, Institute for Mental Health Care Eindhoven (GGzE); 5 University Medical Center Groningen; 6 Maastricht University / Rivierduinen Mental Health Care Institute

## Abstract

**Background:**

For patients with refractory schizophrenia, clozapine is the drug of first choice. However, many refractory patients never receive this drug. The underutilization of clozapine may be caused by the labour-intensive white blood cell monitoring during the first months and the concerns about the safety of outpatient clozapine initiation. A recent survey concluded that professionals “perceived the presence of dedicated staff to arrange and monitor the initiation of clozapine in outpatients as the factor that would enable the use of clozapine most”. We examined whether the presence of such staff in Dutch teams for ambulatory care makes a difference. The primary objective is to examine whether clozapine monitoring by a Nurse Practitioner (NP) is at least as safe as monitoring by a physician. The secondary objective is to examine whether physicians are more likely to prescribe clozapine if they can delegate the monitoring tasks to a NP.

**Methods:**

In this cluster-randomized trial, 23 Dutch ambulatory care teams were randomized into 2 conditions: (A) coordination of clozapine monitoring by a Nurse Practitioner, versus (B) Treatment As Usual: coordination of clozapine monitoring by the responsible physician (usually a psychiatrist). We followed the teams for 15 months, during which period we counted the numbers of patients who started with clozapine. We assessed the safety of the clozapine monitoring by measuring the number of weekly lab exams performed during the first 18 weeks of treatment and counting serious adverse events (SAE). It is important to note that the staff of teams remained blind to the secondary research question.

**Results:**

Of the 2643 patients with a diagnosis of non-affective psychotic disorder, 66 patients started using clozapine during the follow-up, 48 in condition A and 18 in condition B (RR: 2.14, 95% CI: 1.24–3.70; p=.005). The provisional results showed no significant differences between conditions A and B in the mean number of lab exams performed. In condition A, 65% of the mandatory lab exams were carried out compared to 60% in condition B. No agranulocytosis or other SAE occurred in Conditions A or B.

**Discussion:**

Physicians prescribed over 2 times more often clozapine to patients when they could delegate the white blood cell monitoring to a NP. Clozapine-monitoring by an NP appears to be just as safe as monitoring by a physician. These results strongly support the idea that the presence of dedicated staff to arrange and monitor the initiation of clozapine enables the use of this drug.

